# Clinical characterization of polycythemia vera associated with IgA nephropathy in a single Chinese center: A case series

**DOI:** 10.1097/MD.0000000000033493

**Published:** 2022-04-07

**Authors:** Xia Wang, Nannan Yang, Chunyu Lu, Feng Xu, Jinquan Wang

**Affiliations:** a Jinling Hospital, Nanjing University School of Medicine, Nanjing, China; b Jiangsu Province Hospital of Chinese Medicine, Affiliated Hospital of Nanjing University of Chinese Medicine, Nanjing, China; c Nanjing University of Chinese Medicine, Nanjing, China.

**Keywords:** IgA nephropathy, polycythemia vera, prognosis

## Abstract

Polycythemia vera (PV) is a myeloproliferative neoplasm which is characterized by excessive production of erythrocytes as well as myeloid and megakaryocytic proliferation. PV associated with IgA nephropathy (IgAN) has rarely been reported in the literature. The long-term renal prognosis of these patients is unknown.

**Methods:**

Clinical and pathological characteristics of 7 patients with renal biopsy-proven IgAN associated with PV were retrospectively analyzed.

**Results:**

The 7 patients were all males, with a mean age of 49.1 ± 18.8 years when admitted to our hospital. Systemic symptoms include: hypertension in case 2, 3, 5, and 6, splenomegaly in case 2, 4, and 5, and multiple lacunar infarction in case 6. Bone marrow biopsy test revealed relative erythroid hyperplasia and atypical megakaryocyte proliferation which consistent with a chronic myeloproliferative neoplasm. All patients had JAK2V617F and BCR-ABL tested, and JAK2V617F positive in 2 patients. Mild mesangial proliferation was observed in 5 patients and moderate/severe mesangial proliferation in 2patients. Immunofluorescence mainly showed diffuse granular deposition of dominant IgA in mesangium. After follow-up of 56.7 ± 44.0 months, hemoglobin level was 144 ± 29 g/L and hematocrit lever was 0.470 ± 0.03, compared with 187 ± 29 g/L and 0.563 ± 0.087 respectively when admitted to our hospital. The urine protein was 0.85 ± 0.64 g/24 h compared with 3.97 ± 4.68 g/24 h. Case 3 progressed to end stage renal disease and had received hemodialysis for 5 years before renal transplantation.

**Conclusions:**

The results of this study showed that PV associated with IgAN mainly occurs in males and is often accompanied by hematuria and mild-to-moderate renal insufficiency. The long-term prognosis was good for most patients, and few progressed relatively quickly to end stage renal disease.

## 1. Introduction

Myeloproliferative neoplasms is a clonal stem cell disorder, including chronic myeloid leukaemia, polycythemia vera (PV), primary myelofibrosis and essential thrombocythaemia.^[1]^ Polycythemia vera is the most common in Myeloproliferative neoplasms, which characterized by increased erythrocyte production, and identified as a chronic myeloproliferative neoplasm. PV is a rare disease with annual incidence of PV is 0.01 to 2.61 per 100,000 people, and the prevalence is 0.49 to 46.88 per 100,000.^[2,3]^ The incidence of PV is slightly higher in male than female. It is characterized by erythrocytosis, often with associated leukocytosis and thrombocytosis. Other features, including splenomegaly, vasomotor disturbances and pruritus, may occur at any time during the course of PV.^[4]^

The incidence of PV combined with glomerulonephritis is quite low. Including IgA nephropathy, focal segmental glomerular sclerosis, diffuse mesangial proliferative glomerulonephritis, Henoch-Schonlein purpura nephritis may be presented in PV patients.^[5]^ The most common primary glomerular disease in the Chinese population is IgA nephropathy. So far, only 11 cases of PV associated with IgAN have been reported, in which patients presented with proteinuria and hematuria with or without mild renal dysfunction.^[6]^ Here, we retrospectively investigated the clinicopathological features and long-term prognosis of 7 cases of PV associated with IgAN. We will hopefully demonstrate the clinicopathological features and long-term prognosis.

## 2. Methods

We reviewed the renal pathology archives at Jinling hospital, NanJing, from January 2003 to Febuary 2013, and 7 biopsy-proven IgAN combined with history of PV were enrolled in this study. All procedures performed in this study involving human participants were in accordance with the ethical standards of the institutional and/or national research committee and with the 1964 Helsinki Declaration and its later amendments or comparable ethical standards. All patients agreed and signed the informed consent form for the purpose of publication. The diagnosis of PV were established according to the criteria proposed by the World Health Organization 2016 Classification of Tumours of Haematopoietic and Lymphoid Tissue.^[7]^ The diagnostic criteria of IgAN included IgA deposition or predominant IgA deposition in the glomerular mesangium as shown by immunofluorescence microscopy.^[8]^ Patients with Secondary IgAN were not included.

Each patient was subjected to detailed demographic information, and underwent clinical examination (including renal biopsy, the routine examination, biopsy of bone marrow biopsy and JAK2V617F).

## 3. Results

The study consisted of 7 men with a mean age of 49.1 ± 18.8 years at the time of renal biopsy. Case 1 and 4 were diagnosed with PV and IgAN at the same time. Case 3, 5, and 6 were diagnosed with PV before the kidney damage. Case 2 and 7 were diagnosed with PV followed by IgAN. The history of hypertension was presented in case 2, 3, 5, 6, multiple lacunar infarction was presented in case 6, and splenomegaly was presented in case 2, 4, 5. When the patients were admitted to our hospital, their chief complaints were proteinuria and microscopic hematuria. Heavy proteinuria was found in case 1 and 2. All the patients except case 1 had microscopic hematuria. Mean serum creatinine levels were 1.17 ± 0.36 mg/dL. Mean blood platelet levels were 332 × 109 ± 78 × 10^9^g/L, hemoglobin levels were 187 ± 29 g/L and hematocrit levels were 56.3 ± 8.7% on diagnosis of PV (Table 1). Histologically, all the patients performed bone marrow biopsy test, which revealed relative erythroid hyperplasia and atypical megakaryocyte proliferation consistent with a chronic myeloproliferative neoplasm. All cases performed the test of JAK2V617F and BCR-ABL. The mutant allele leading to a valine to phenylalanine substitution at amino acid 617 (V617F) of the JAK2 tyrosine kinase was found in case 3 and 4 (Table 2).

**Table 1:** Clinical data of patients with polycythemia vera associated with IgAN at renal biopsy.

Patient	1	2	3	4	5	6	7
Sex	M	M	M	M	M	M	M
Age at biopsy (yr)	22	40	55	33	70	73	51
PV duration (mo)	31	30	61	16	358	144	48
Renal disease duration (mo)	31	32	50	16	83	108	216
Hypertension	No	Yes	Yes	No	Yes	Yes	No
Splenomegaly	No	Yes	No	Yes	Yes	No	No
Hemoglobin (g/L)	186	223	210	201	149	158	175
PLT (10^9^/L)	250	256	333	439	410	306	341
WBC (10^9^/L)	6.2	10.9	9.2	9.7	8.9	5.2	5.4
HCT (%)	55.7	59.9	67	62.8	44.5	47.9	51.4
Proteinuria (g/24 h)	11.76	9.63	2.1	1.25	2.06	0.73	0.25
Urinary microscopic hematuria (10,000 cells/mL)	1	570	95	72	12	22	10
Serum albumin (g/L)	23.2	31.4	38.5	40.8	45.7	46.5	46
Serum creatinine (mg/dL)	1.09	1.05	1.91	0.75	1.19	1.28	0.95
eGFR (mL/min/1.73 m^2^) at biopsy	98.89	84.04	37.95	130.77	65.35	59.3	90.99
Bone marrow	+	+	+	+	+	+	+
JAK2V617F	−	−	+	+	ND	−	ND
BCR-ABL	−	−	−	−	ND	−	ND

HCT = hematocrit, M = male, PLT = platelet, pv = polycythemia vera, WBC = white blood cell count.

**Table 2:** Pathological observations in patients with polycythemia vera associated with IgA nephropathy.

Patient	Light microscopy							Immunoflurescent study				Electron microscopy
	No. of glomeruli	% of globally sclerotic glomeruli	% of segmentally sclerotic glomeruli	No. of crescents	Mesangial proliferation	Tubolo interstitial lesion	Vessel lesion	IgA	IgM	IgG	C3	Electron-dense material
1	32	3.1	0	0	No	Mild	No	++	−	−	++	Yes
2	15	40	33.3	1	Moderate/Severe	Moderate	Yes	++	+	++	++	Yes
3	26	38.4	7.6	0	Mild	Moderate	Yes	++	+	−	−	Yes
4	20	5	0	0	Mild	Mild	Yes	++	−	−	+	Yes
5	ND	ND	Yes	0	Mild	Moderate	Yes	+++	−	−	+	ND
6	ND	ND	Yes	0	Moderate	Moderate	No	++	+-	−	+	Yes
7	ND	ND	No	0	Mild	Mild	No	+++	−	−	++	ND

ND = not presented/not done.

The renal biopsy showed mild mesangial proliferation in 5 patients, moderate/severe mesangial proliferation in 2 patients. Tubules were mildly atrophic and epithelial cells displayed degeneration or regeneration. Immunofluorescence microscopy revealed diffuse granular deposition of dominant IgA in mesangium with less amount of C3. Ultrastructurally, mesangial and paramesangial electron-dense deposits with the mesangial matrix widening were observed.

Before admission, case 3, 5, and 6 were diagnosed with PV in other hospitals, and case 3 and 5 received hydroxycarbamide, case 6 received interferon after diagnosed. Case 3 and 6 received ACEI/ARBs at the same time. After admission to Jinling hospital and diagnosed with IgAN by renal biopsy, case 2, 3, 5, and 7 received Tripterygium wilfordii, case 1 and 2 were also received prednisone. Case 1 and 4 received hydroxycarbamide. During the follow-up, case 2 and 7 were diagnosed with PV and received hydroxycarbamide. After follow-up of 56.7 ± 44.0 months (ranging from 14–144 months), mean blood platelet levels were 416 × 10^9^ ± 194 × 10^9^g/L, hemoglobin levels were 144 ± 29 g/L and hematocrit levels were 47 ± 3%. The urine protein was 0.85 ± 0.64 g/24 h compared with 3.97 ± 4.68 g/24 h at the time of biopsy. Case 3, 5, and 7 showed persistent renal dysfunction and case 3 progressed to end stage renal disease (ESRD) and had received hemodialysis for 5 years before renal transplantation (Table 3).

**Table 3:** The outcome of the 7 patients.

Patient	1	2	3	4	5	6	7
Hemoglobin (g/L)	166	155	83	160	132	151	163
PLT (10^9^/L)	222	224	557	554	724	314	322
HCT (%)	50.5	47	49.5	49.1	40.7	43.9	48
Proteinuria (g/24 h)	0.12	0.81	1.8	0.52	1.66	0.81	0.29
Urinary microscopic hematuria (10,000 cells/mL)	1	4	127	2	32	16	3
Serum albumin (g/L)	47.7	40.9	54.2	49.1	43.1	43.3	43.7
Serum creatinine (mg/dL)	1.02	1.17	8.19	0.75	3.53	1.14	1.35
eGFR (mL/min/1.73 m^2^)	95.31	73.53	6.3	130.77	17.08	68.41	58.98
Treatment	P + HU + ARB	P + TW + LEF + HU + ARB	TW + HU + ACEI	HU + IFN + ARB	TW + HU	IFN + ARB	TW + HU
Follow-up from biopsy (m)	31	30	48	14	82	48	144

ACEI = angiotensin-converting enzyme inhibitor, ARB = angiotensin receptor blocker, CR = complete remission, HCT = hematocrit, HU = hydroxycarbamide, IFN = interferon, KT = kidney transplantation, LEF = leflunomide, P = prednisone, PLT = platelet, PRD = persistent renal dysfunction, TW = tripterygiumwilfordii.

## 4. Discussion

Polycythemia vera is a myeloproliferative neoplasm of unknown etiology that involves the clonal proliferation of erythrocytes.^[9]^ IgAN is recognized as the most common glomerulonephritis worldwide, and it can be combined with other diseases. PV associated with renal disease is clinically rare. To the best of our knowledge, only 11 cases of PV associated with IgAN have been reported in the literature.^[6,10–16]^ The 11 patients were 10 males and 1 female with a mean age of 45.1 ± 12.6 years. 7 cases (58.3%) presented nephrotic syndrome, 3 case (25%) presented moderate proteinuria. 9 cases (75%) with microscopic hematuria, and 2 cases (16.7%) were negative. Hypertension and nephrotic syndrome-range proteinuria (58.3%) were most common clinical presentation reported in the literature. With respect to patient follow-up, 1 case (8.3%) progressed to ESRD and required routine dialysis. In the remaining 3 cases (25%), proteinuria was complete remission. In our study, 4 cases (57%) were presented with hypertension, 2 cases (29%) were presented with nephrotic syndrome-range proteinuria, and 5 cases (71%) were presented with proteinuria. The renal insufficiency was common presentation reported in the literature. The renal outcome in our study was followed up 56.7 ± 44.0 months. Case 3 reached ESRD, whose renal histological biopsy showed 38.4% global and 7.6% segmental glomerular sclerosis. The Proteinuria was completely remitted in case 1 and 7, proteinuria and renal dysfunction were effectively controlled in case 2, 4, and 5, and proteinuria remained stable in case 6. During the follow-up period, there were significant differences in hemoglobin levels and hematocrit levels.

The pathogenesis of PV associated with IgAN is still unclear. Cytokines and growth factors, such as platelet-derived growth factor (PDGF) and transforming growth factor (TGF)-β, may play important roles in the pathogenesis in renal disease associated with PV. PDGF is the most potent stimulus of mesangial cell proliferation, and it also induces extracellular matrix produced by mesangial cells.^[17,18]^ Transforming growth factor (TGF)-β induces mesangial sclerosis by enhancing the synthesis of collagen and fibronectin of mesangial cells,^[19,20]^ and has pro-apoptotic podocyte effects, which may promote podocyte depletion and the focal segmental glomerular sclerosis lesions seen in most cases.^[21]^ Growth factors produced by marginating platelets and cells of extramedullary hematopoiesis together with hyperviscosity aggravate the endothelial damage.^[22]^

Erythropoiesis is one of the features of PV. According to a recent large cohort study of chronic kidney disease, IgAN was an independent factor associated to higher hemoglobin levels, and higher risk of developing erythrocytosis.^[23]^ The pathogenesis of IgAN includes the aberrant alactosylation of polymeric IgA1 in the hinge region,^[24]^ which can stimulate erythropoiesis in vitro and in vivo in IgA1 humanized mice, and results in elevated serum levels of galactose-deficient polymeric IgA1.^[25]^ Evidence suggests that polymeric IgA1 might be a regulator of erythropoiesis in humans.^[26]^ These IgA1 increased the sensitivity of erythroid progenitors to erythropoietin in vitro, possibly explaining the pathophysiology of PV combined with IgAN.

Based on the present literatures, the other possible pathogenesis of PV associated with renal disease also include changing blood volume and viscosity which resulted in mesangial hypercellularity and matrix proliferation, vascular microthrombi, interstitium ischemia, and more rapid progression of renal failure.^[27,28]^ However, according to the literature, the coexistence of the 2 diseases might be simply coincidental and no causative relationship is presented.

The present study has limitations, arising from the retrospective nature and small number of enrolled patients. We were unable to assess the incidence of PV associated with IgAN. Additional JAK2 mutations in exon 12 were detected in a subset of JAK2V617F-negative patients with PV.^[29,30]^ However, the patients in our study didn’t perform the test. And we also unable to determine the serum levels of growth factors.

## 5. Conclusion

The clinical characteristics of PV associated with IgAN were common in males. Nephrotic-range proteinuria and renal insufficiency as well as histologically changes (variable degrees of mesangial proliferation and segmental sclerosis) were always found in these patients. The longterm prognosis is good for most patients.

## Author contributions

**Conceptualization:** Jinquan Wang.

**Investigation:** Nannan Yang, Chunyu Lu.

**Methodology:** Feng Xu.

**Project administration:** Jinquan Wang.

**Writing – original draft:** Xia Wang.

**Writing – review & editing:** Jinquan Wang.
